# Prognostic significance of blood-based PD-L1 analysis in patients with non-small cell lung cancer undergoing immune checkpoint inhibitor therapy: a systematic review and meta-analysis

**DOI:** 10.1186/s12957-023-03215-2

**Published:** 2023-10-11

**Authors:** Qian Cui, Wentao Li, Dong Wang, Shuangcui Wang, Jianchun Yu

**Affiliations:** 1 Department of Oncology, First Teaching Hospital of Tianjin University of Traditional Chinese Medicine, Tianjin, China; 2 National Clinical Research Center for Chinese Medicine Acupuncture and Moxibustion, Tianjin, China; 3 Graduate School, Tianjin University of Traditional Chinese Medicine, Tianjin, China

**Keywords:** Non-small cell lung cancer, Liquid biopsy, Blood-based PD-L1, Meta-analysis

## Abstract

**Background:**

The main types of PD-L1 in the blood include soluble PD-L1 (sPD-L1), exosomal PD-L1 (exoPD-L1), and PD-L1 in circulating tumor cells (CTCs). However, the predictive and prognostic values of these three indicators in patients with non-small cell lung cancer (NSCLC) undergoing immune checkpoint inhibitor (ICI) therapy are unclear, warranting a systematic meta-analysis.

**Methods:**

A systematic literature search was performed in the PubMed, Cochrane Library, and Embase databases. The pooled hazard ratio (HR) and 95% confidence interval (CI) values were extracted from the included studies to investigate the correlation between the three PD-L1 indicators and overall survival (OS) or progression-free survival (PFS). The Newcastle–Ottawa Scale (NOS) was used to examine the quality of the included studies. Subgroup analyses were employed to investigate the heterogeneity. The publication bias of the included studies was assessed using Begg's and Egger's tests. *P* < 0.05 was regarded as significantly different.

**Results:**

The pooled results revealed that high pre-treatment sPD-L1 levels were significantly associated with inferior OS (HR = 2.32, 95% CI = 1.68–3.18, *P* < 0.001) and PFS (HR = 2.52, 95% CI = 1.72–3.68, *P* < 0.001). However, dynamic changes in sPD-L1 after immunotherapy were not statistically significant for OS (HR = 1.46, 95% CI = 0.65–3.26, *P* > 0.05) or PFS (HR = 1.62, 95% CI = 0.92–2.86, *P* > 0.05). Meanwhile, the upregulated pre-treatment exoPD-L1 levels were significantly associated with poor PFS (HR = 4.44, 95% CI = 2.87–6.89, *P* < 0.001), whereas the post-treatment dynamic upregulation of exoPD-L1 was significantly correlated with superior PFS (HR = 0.36, 95% CI = 0.24–0.54, *P* < 0.001) and OS (HR = 0.20, 95% CI = 0.07–0.53, *P* < 0.001). For PD-L1 in CTCs, the pooled results indicated that PD-L1 expression in CTCs was not significantly correlated with OS (HR = 0.75, 95% CI = 0.49–1.13, *P* = 0.170) and PFS (HR = 0.79, 95% CI = 0.59–1.06, *P* = 0.12).

**Conclusions:**

Blood-based PD-L1 analysis is a potential strategy for predicting treatment efficacy and prognosis in patients with cancer.

**Supplementary Information:**

The online version contains supplementary material available at 10.1186/s12957-023-03215-2.

## Background

Among the malignant tumors, lung cancer, a global public health issue, is the most frequent cause of tumor-related deaths globally [1]. Small cell lung cancer and non-small cell lung cancer (NSCLC) are the two types of lung cancer based on their histology, with NSCLC accounting for about 85% of all lung cancer cases [1]. Although there have been recent breakthroughs in the diagnosis and treatment of cancer, the overall 5-year survival rate for all NSCLC patients remains at 24.6%. Patients with stage I and stage IV have 5-year survival rates of 65% and 5%, respectively [2].

Immune checkpoint inhibitors (ICIs) have improved the survival outcomes of patients with cancer [3]. The US Food and Drug Administration recently approved ICIs to manage NSCLC [4]. Currently, ICIs serve as the first-line and second-line treatments for NSCLC [5, 6]. ICIs have been developed to target immune checkpoint molecules, such as programmed cell death protein 1 (PD-1) and programmed cell death ligand 1 (PD-L1), which are critical for regulating anti-tumor immune responses [7]. The interaction of PD-L1 with PD-1 suppresses T-cell activity and proliferation, resulting in immunological tolerance. Pembrolizumab, an anti-PD-1 antibody, is used to treat patients with NSCLC exhibiting high PD-L1 expression in tumor tissue [8]. However, clinical studies have reported that only 15%–30% of patients achieve favorable outcomes with ICIs [9]. Some patients experience immune-related adverse reactions [10]. Therefore, it is essential to identify biomarkers to assess treatment efficacy and disease prognosis, as well as to screen patients who will benefit from ICI therapy. Tissue PD-L1 (tPD-L1) expression is the most common biomarker for assessing the efficacy and prognosis of ICIs in NSCLC [11]. Previous studies have suggested that highly expressed tPD-L1 improves the clinical prognosis of patients with tumors undergoing ICI therapy [12, 13]. However, recent studies have reported that several individuals with high tPD-L1 expression do not respond to immunotherapy. In contrast, some patients with low tPD-L1 expression had long-term efficacy [14]. A recent meta-analysis revealed that tPD-L1 alone is not an appropriate molecular biomarker for identifying eligible patients for immunotherapy in routine clinical practice [15]. Furthermore, the detection of PD-L1 involves tissue sampling from patients with cancer. Repeated tissue biopsy analysis is challenging for patients with advanced lung cancer. Cancer develops over time due to dynamic molecular changes and harbors escape mutations and epigenetic modifications [16, 17]. This can result in therapy resistance and disease progression, which cannot be evaluated using a single biopsy.

The use of blood-based biopsies to analyze PD-L1 expression in patients with cancer can overcome the challenges associated with the use of solid tumor biopsies. Compared with tissue biopsies, blood-based biopsy techniques have several advantages, including ease of sampling and reproducible monitoring of the dynamic biological changes in vivo [18]. Some studies have reported that tumor cells and immune cells can both release PD-L1 into the bloodstream [19]. Recent studies have revealed that three blood-based PD-L1 indicators, namely soluble PD-L1 (sPD-L1), exosomal PD-L1 (exoPD-L1), and PD-L1 in circulating tumor cells (CTCs), are linked to prognosis in patients with cancer [20–22]. Several studies have shown that the elevated pre-treatment sPD-L, PD-L1 in CTCs, and exoPD-L1 levels are negatively correlated with the prognosis of patients with NSCLC undergoing immunotherapy [23–25]. The dynamic upregulation of exoPD-L1 and positive PD-L1 expression in CTCs (PD-L1^+^ CTCs) after immunotherapy were significantly and positively correlated with progression-free survival (PFS) [26, 27]. However, Tiako Meyo et al. reported that upregulated post-treatment levels of sPD-L were not significantly correlated with overall survival (OS) [28]. The roles of these three blood-based PD-L1 indicators in determining the prognosis of patients with NSCLC before and after immunotherapy are unclear. Thus, this study performed a meta-analysis to investigate if the three blood-based PD-L1 indicators are correlated with the prognosis of patients with NSCLC undergoing ICI therapy.

## Methods

### Protocol and registration

The recommended reporting criteria for the Preferred Reporting Items for Systematic Reviews and Meta-Analysis (PRISMA) guidelines were followed to perform the meta-analysis. The study protocol was prospectively registered at PROSPERO (CRD42023402077) and is available at https://www.crd.york.ac.uk/prospero/display_record.php?ID=CRD42023402077.

### Search strategy

Relevant literature in the English language published up to February 23, 2023, was retrieved from the PubMed, Embase, and Cochrane Library databases. The following keywords were used: "non-small cell lung cancer," "lung cancer," "lung tumor," "lung neoplasm," "immune checkpoint inhibitor," "PD-1," "PD-L1," "anti-PD-1," "PD-L1," "programmed death 1 receptor," "programmed death ligand 1," "cytotoxic T lymphocyte antigen 4," "CTLA-4," "blood," "circulating tumor cell," "PD-L1," "soluble PD-L1," "exosomal PD-L1," "circulating PD-L1," "PD-L1 in CTCs," "sPD-L1," and "exoPD-L1." The references in the reviews were manually searched to identify additional related articles.

### Inclusion criteria

Studies on patients with pathologically diagnosed primary or metastatic NSCLC; studies on patients undergoing ICI mono-therapy or ICI com-therapy (chemotherapy or targeted therapy); studies evaluating the correlation between PD-L1-based blood markers (PD-L1 in CTCs, sPD-L1, and exoPD-L1) and OS/PFS of patients with NSCLC; studies reporting the hazard ratio (HR) and 95% confidence interval (CI). If the HR was not directly available from studies, it was extracted from the Kaplan–Meier curve. When the univariate and multivariate analysis results were provided simultaneously, only the multivariate analysis results were extracted.

### Exclusion criteria

Case reports, reviews, meta-analyses, conference abstracts, and letters; studies in which the full text was not available for download; studies that included animal experiments; studies in non-English language.

### Quality assessment

Based on selection, comparability, and outcomes, the quality of the included studies was evaluated using the Newcastle–Ottawa scale (NOS). The NOS quality score ranged from 0 to 9. Studies with NOS scores of ≥ 7 and < 7 were categorized as high-quality and low-quality studies, respectively [29].

### Data extraction

Information, such as the name of the first author, the publication year, the region, the sample size, the age, the gender, the cancer stage, the cut-off value, the time point, the study design, and the outcome, was retrieved from each included study. Two authors (Qian Cui and Dong Wang) independently extracted data from univariate and multivariate analyses with HR and 95% CI for OS or PFS. Any controversy was addressed via discussion.

### Statistical analysis

The HR and 95% CIs were extracted to assess the association between these three blood-based PD-L1 indicators and survival outcomes of patients with NSCLC undergoing ICI therapy. Additionally, the association between clinicopathologic features and survival outcomes was evaluated by the HR and 95% CI. The HR values of > 1 and < 1 were considered adverse and favorable prognoses, respectively. Cochran's q-test and *I*^*2*^ combined values were applied to analyze the heterogeneity of the results [30]. *P* > 0.10 and *I*^*2*^ < 50% indicated low-level heterogeneity [29]. Subgroup analysis was utilized to investigate the heterogeneity of the studies. Furthermore, Begg's and Egger's tests were employed to assess publication bias. Differences were considered significant at *P* < 0.05. All statistical analyses were performed using Stata 15.1 software.

The HR and 95% CIs were extracted to assess the association between these three blood-based PD-L1 indicators and survival outcomes of patients with NSCLC undergoing ICI therapy. Additionally, the association between clinicopathologic features and survival outcomes were evaluated by the HR and 95% CI.

## Results

### Literature screening

**Fig. 1:**
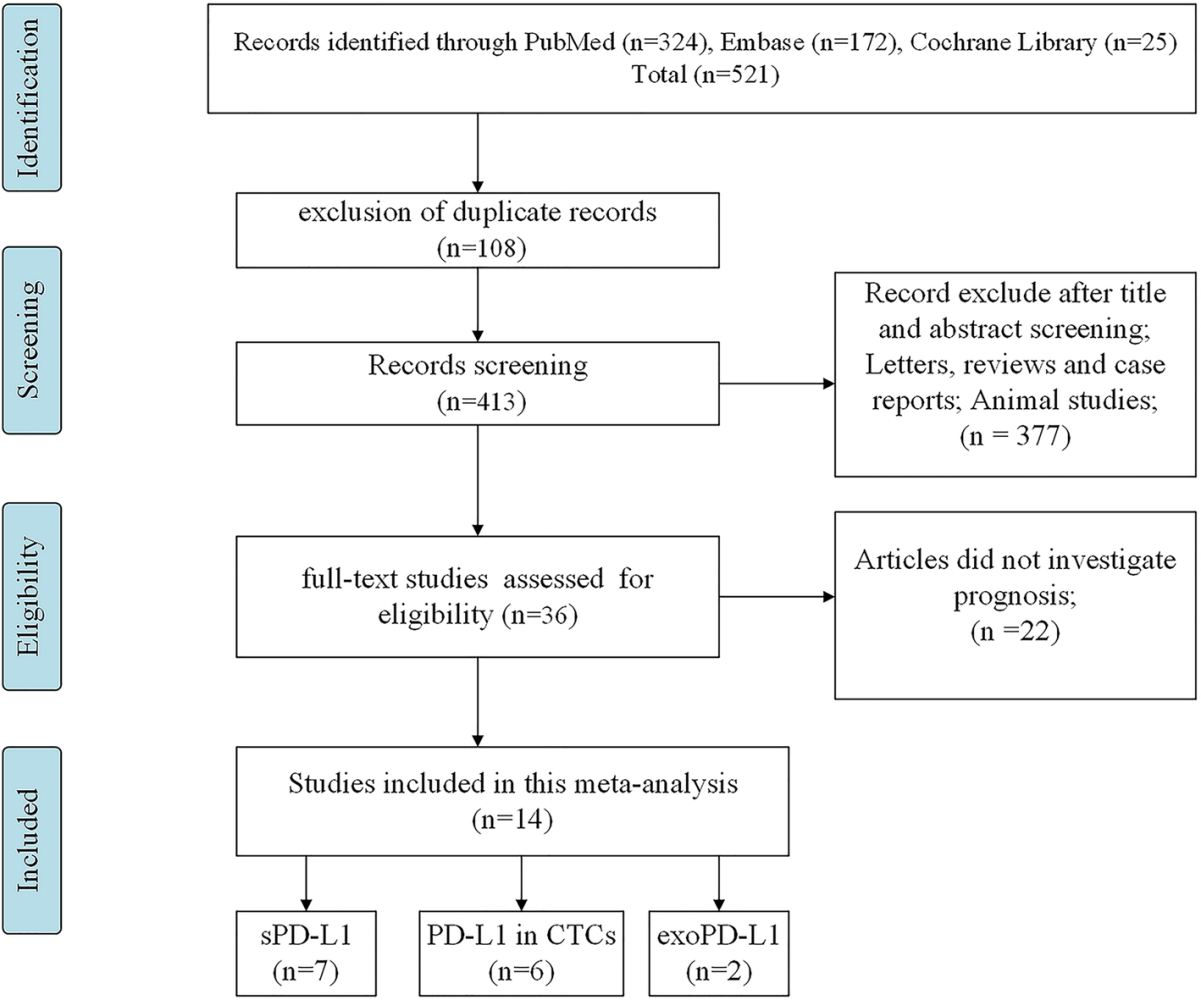
PRISMA flowchart for screening the literature

Literature searched in the PubMed, Embase, and Cochrane Library databases yielded 521 studies. Of these, 490 studies were excluded as they were duplications, animal studies, commentaries, and incomplete full texts. The complete texts of the remaining 36 articles were comprehensively evaluated. Of these, 22 studies were excluded, and 14 studies were included in this meta-analysis [23–28, 31–38]. The flow chart is illustrated in Fig. 1.

### Study characteristics and quality evaluation

As shown in Table 1, the analysis included 14 trials performed between 2018 and 2022 involving 928 patients with NSCLC. Of these, seven, six, and one studies were performed in Asia, Europe, and America, respectively. Most of the published studies were prospective studies, while only one was a retrospective study. Among the studies included in the meta-analysis, seven, six, and two studies evaluated sPD-L1, PD-L1 in CTCs, and exoPD-L1, respectively. Regarding treatment duration, the pre-treatment sPD-L1 levels were evaluated in six studies, while the dynamic changes in the post-treatment sPD-L1 levels were evaluated in only two studies. Meanwhile, the pre-treatment and post-treatment PD-L1 levels in CTCs were evaluated in four and two studies, respectively. Furthermore, two studies evaluated the pre-treatment exoPD-L1 levels, while one study evaluated dynamic changes in the post-treatment exoPD-L1 levels. The NOS quality score was ≥ 7 and < 7 for 12 and 2 studies, respectively.

**Table 1:** Baseline features of 14 studies

Author	Year	Region	Sample size	Age (median/range)	Gender (male/ female)	Stage	Indexes	Cut-off values	Study design	Outcome	Time point	NOS score
Mazzaschi [23]	2020	Italy	109	72 (41–85)	73/36	IIIB–IV	sPD-L1	113 pg/ml	P	PFS/OS	Pre	8
Wang [24]	2022	China	149	-	119/30	II–IV	exoPD-L1	0.54 pg/ml	P	PFS	Pre	8
Guibert [25]	2018	France	96	-	-	IV	PD-L1 in CTCs	1%/ 5%/10%	P	PFS/OS	Pre	7
Yang [26]	2021	China	21	60	-	Advanced	sPD-L1/exoPD-L1	-	P	PFS/OS	Post	7
Dall'Olio [27]	2021	Italy	39	68	24/15	Advanced	PD-L1 in CTCs	1	P	PFS/OS	Post	8
Tiako Meyo [28]	2020	France	87	66 (60–69)	29/22	IV	sPD-L1	0.156 ng/ml	P	PFS/OS	Pre/Post	8
Costantini [31]	2018	France	43	-	29/14	I–IV	sPD-L1	33.97 pg/ml	P	PFS/OS	Pre/Post	7
Okuma [32]	2018	Japan	39	75	29/10	IV/ recurrent	sPD-L1	3.357 ng/ml	p	OS	Pre	6
Murakami [33]	2020	Japan	233	63 (30–84)	152/81	Advanced	sPD-L1	90 pg/ml	R	PFS/OS	Pre	7
Zizzari [34]	2020	Italy	22	65	16/6	IV	sPD-L1	-	P	OS	Pre	7
Dhar [35]	2018	America	22	67	13/9	IV	PD-L1 in CTCs	2	P	PFS	Pre	7
Zhang [36]	2020	China	16	-	-	III–IV	PD-L1 in CTCs	-	P	PFS	Post	6
Ikeda [37]	2021	Japan	44	68 (49–86)	33/11	III–IV	PD-L1 in CTCs	7.7%	P	PFS/OS	Post	7
Zhang [38]	2022	China	30	56 (48–69)	22/8	III–IV	PD-L1 in CTCs	32.5%	P	PFS/OS	Pre	7

*Aberrations*: *P* Prospective studies,*R* Retrospective studies, *Pre* Pre-treatment, *Post* Post-treatment (Dynamic changes); *OS* Overall survival, *PFS* Progression-free survival

### Correlation between sPD-L1 and prognosis of patients with NSCLC undergoing ICI therapy

**Fig. 2:**
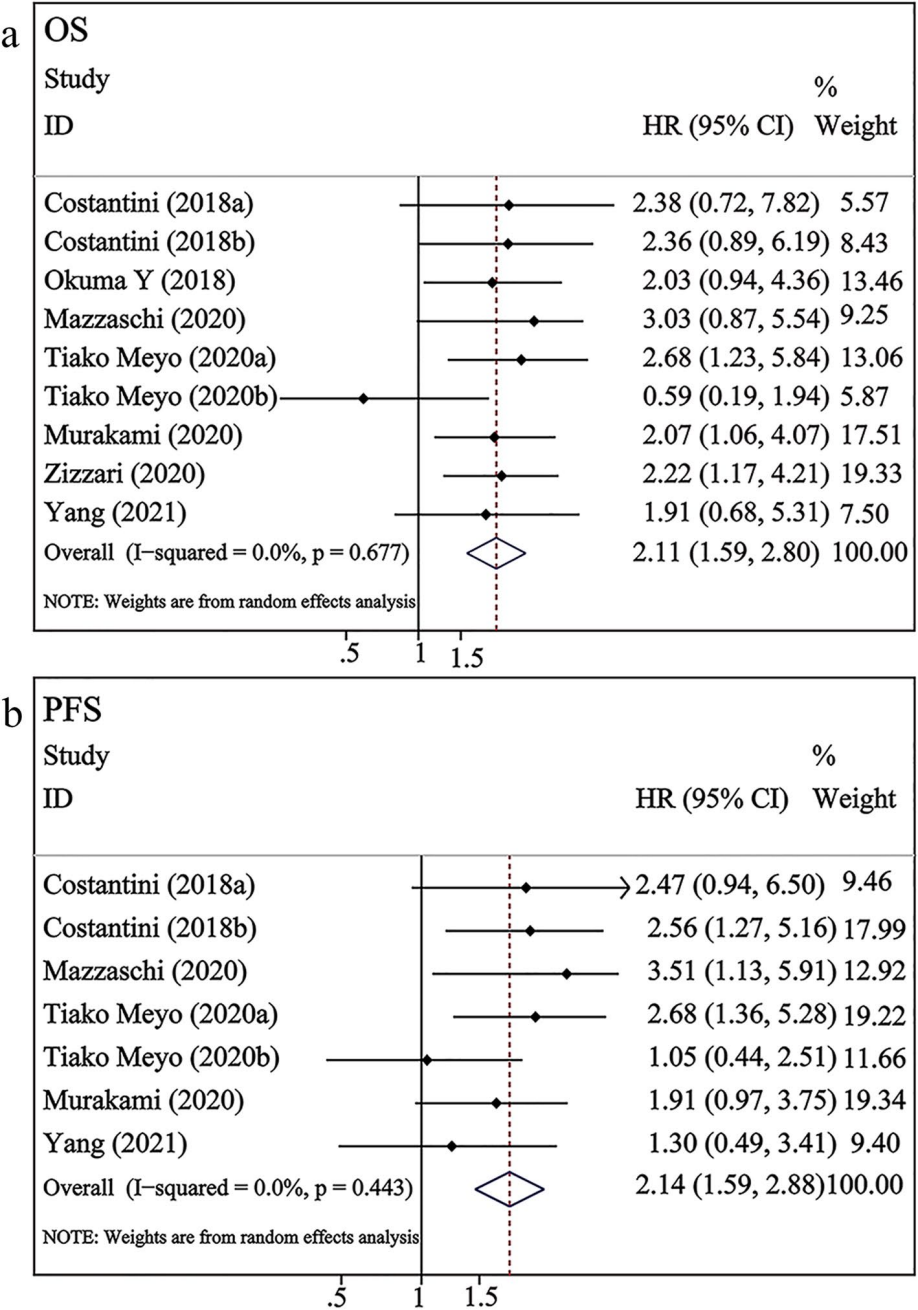
Forest plots of sPD-L1 versus OS/PFS in NSCLC patients undergoing ICI therapy. a–b, sPD-L1 expression versus OS/PFS; Costantini (2018a) and Tiako Meyo (2020a) examined the pre-treatment levels of sPD-L1; Costantini (2018b) and Tiako Meyo (2020b) examined the dynamic changes in the post-treatment sPD-L1 levels

As shown in Fig. 2a, seven studies involving 554 patients with NSCLC undergoing ICI therapy examined the correlation between sPD-L1 and OS. The pooled results revealed that high levels of sPD-L1 were significantly correlated with worse OS (HR = 2.11, 95% CI 1.59–2.80, *P* < 0.001), and a significant heterogeneity was not observed among studies (*I*^*2*^ = 0%, *P* = 0.677). As shown in Fig. 2b, five studies involving 493 patients with NSCLC undergoing ICI therapy revealed the correlation between sPD-L1 and PFS. The pooled results demonstrated that high blood levels of sPD-L1 were negatively correlated with PFS (HR = 2.14, 95% CI 1.59–2.88, *P* < 0.001), and these studies had low-level heterogeneity (*I*^*2*^ = 0%, *p* = 0.443).

**Fig. 3:**
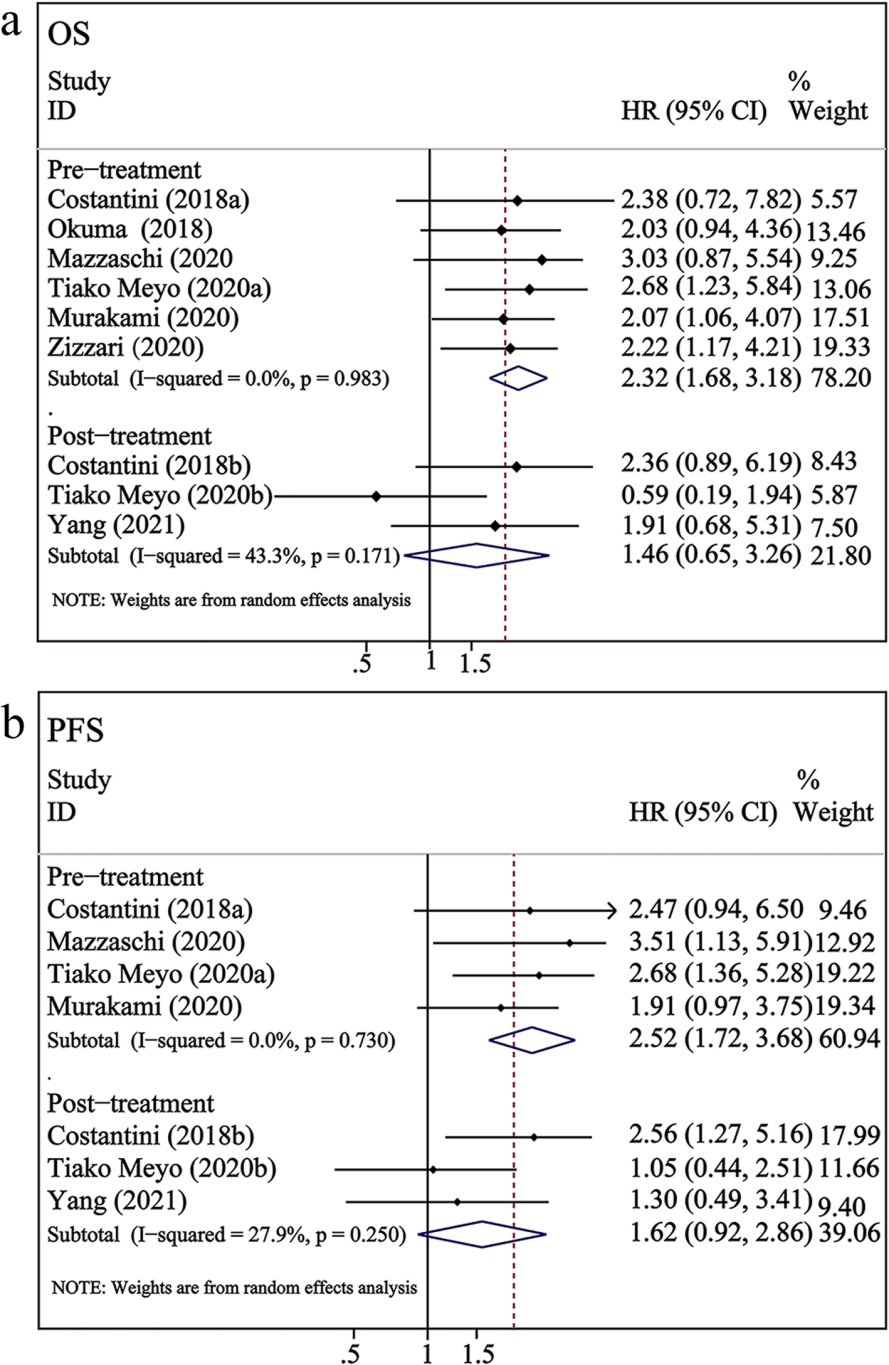
Subgroup analysis of sPD-L1 levels versus OS/PFS after different treatment durations

Subgroup analysis was performed based on the correlation of sPD-L1 with OS and PFS according to the time point, sample size, and region. No significant heterogeneity was observed in most subgroups (Table 2). Stratified analysis revealed that high pre-treatment levels of sPD-L1 were remarkably correlated with poorer OS (HR = 2.32, 95% CI 1.68–3.18, *P* < 0.001; Table 2, Fig. 3a) and PFS (HR = 2.52, 95% CI 1.72–3.68, *P* < 0.001; Table 2, Fig. 3b). However, after immunotherapy, the dynamic upregulation of sPD-L1 levels was not significantly linked to OS (HR = 1.46, 95% CI = 0.65–3.26, *P* > 0.05; Table 2, Fig. 3a) and PFS (HR = 1.62, 95% CI = 0.92–2.86, *P* > 0.05; Table 2, Fig. 3b). Furthermore, moderate heterogeneity was observed in the subgroups of post-treatment sPD-L1 and OS (*I*^*2*^ = 43.3%, *p* = 0.171).

**Table 2:** Subgroup analysis of blood-based PD-L1 markers and OS/PFS in NSCLC receiving ICIs

Subgroup	N	HR (95% CI)	Interaction	Heterogeneity	
**OS**			***P***	**I**^**2**^**%, *P***	***P* interaction**
**sPD-L1**					
Total	9	2.11 (1.59–2.80)	< 0.001	0%, 0.677	
**Time point**					0.295
Pre-treatment	6	2.32 (1.68–3.18)	< 0.001	0%, 0.983	
Post-treatment	3	1.46 (0.65–3.26)	0.360	43.3%, 0.171	
**Sample size**					0.631
> 100	2	2.36 (1.37–4.07)	< 0.01	0%, 0.514	
≤ 100	7	2.02 (1.46–2.81)	< 0.001	0%, 0.533	
**Region**					0.890
Asia	2	2.05 (1.24–3.40)	< 0.01	0%, 0.970	
Europe	7	2.14 (1.52–3.00)	< 0.001	0%, 0.456	
**PD-L1 in CTCs**					
Total	6	0.75 (0.49–1.13)	0.170	33.5%, 0.185	
**Time point**					0.802
Pre-treatment	5	0.73 (0.44–1.21)	0.222	46.3%, 0.114	
Post-treatment	1	0.83 (0.35–1.98)	0.674	-	
**Sample size**					0.156
> 30	5	0.84 (0.54–1.30)	0.426	25.2%, 0.254	
≤ 30	1	0.43 (0.19–0.97)	< 0.05	-	
**Region**					0.415
Asia	2	0.59 (0.31–1.12)	0.104	14.6%, 0.279	
Europe	4	0.84 (0.48–1.46)	0.529	43.9%, 0.148	
**PFS**					
**sPD-L1**					
Total	7	2.14 (1.59–2.88)	< 0.001	0%, 0.443	
**Time point**					0.250
Pre-treatment	4	2.52 (1.72–3.68)	< 0.001	0%, 0.730	
Post-treatment	3	1.62 (0.92–2.86)	0.097	27.9%, 0.250	
**Sample size**					0.562
> 100	2	2.47 (1.37–4.44)	< 0.01	19.8%, 0.264	
≤ 100	5	2.01 (1.38–2.91)	< 0.001	5.5%, 0.375	
**Region**					0.726
Asia	1	1.91 (0.97–3.75)	0.061	-	
Europe	6	2.19 (1.53–3.12)	< 0.001	12.1%, 0.338	
**PD-L1 in CTCs**					
Total	8	0.79 (0.59–1.06)	0.12	20.6%, 0.266	
**Time point**					0.530
Pre-treatment	6	0.75 (0.52–1.08)	0.124	36.8%, 0.161	
Post-treatment	2	0.95 (0.50–1.80)	0.882	0%, 0.449	
**Sample size**					0.754
> 30	5	0.82 (0.57–1.17)	0.267	30.0%, 0.222	
≤ 30	3	0.73 (0.39–1.38)	0.332	30.6%, 0.237	
**Region**					0.924
Asia	3	0.71 (0.39–1.27)	0.248	29.1%, 0.244	
Europe	4	0.82 (0.53–1.26)	0.368	46.5%, 0.132	
America	1	0.83 (0.24–2.86)	0.768	-

### Correlation between CTC-PD-L1 and prognosis in patients with NSCLC undergoing ICI therapy

**Fig. 4:**
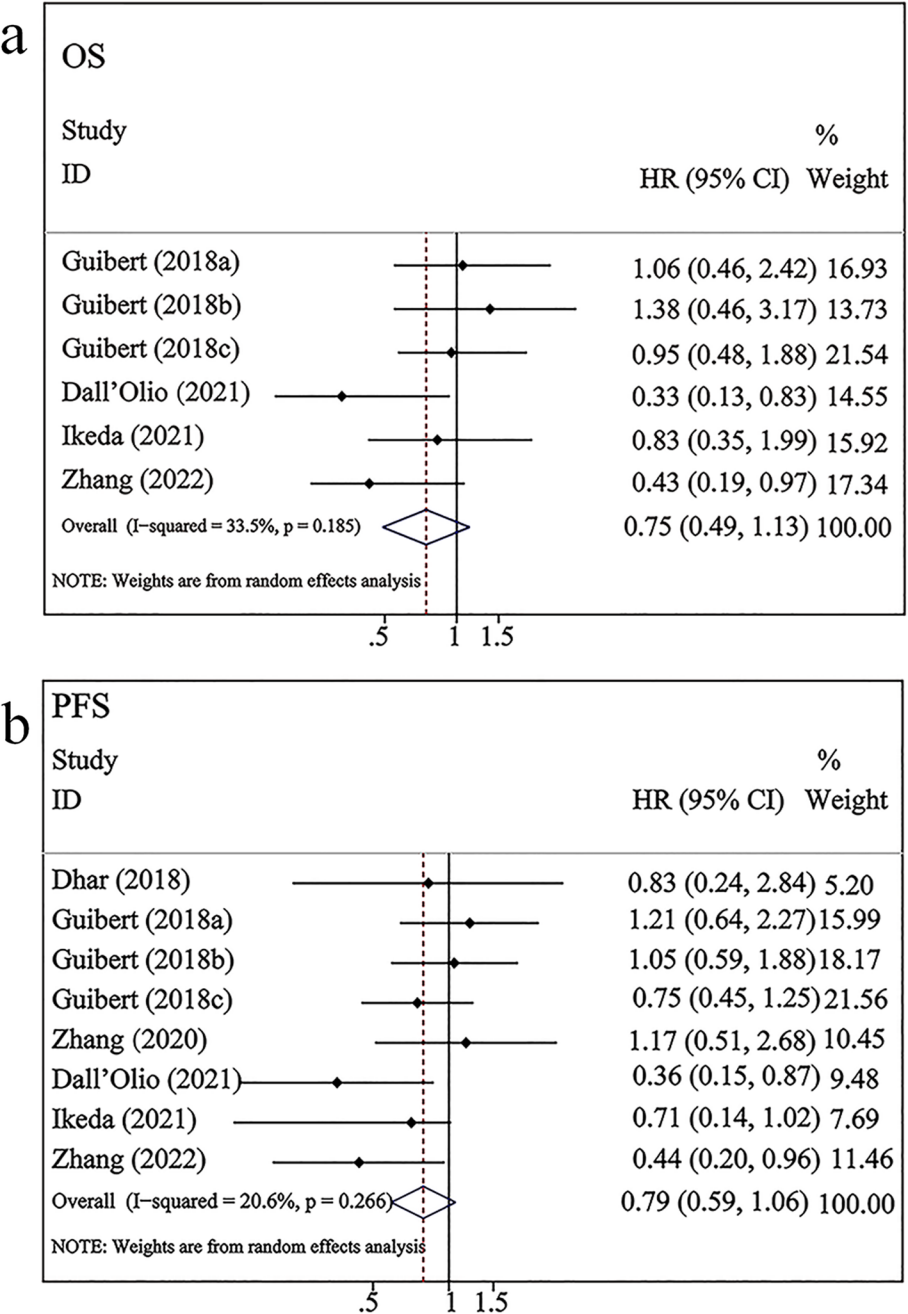
Forest plots of PD-L1 expression on CTCs and OS/PFS in patients undergoing ICI therapy. a–b: PD-L1 expression in CTCs versus OS/PFS; Guibert (2018a) represented the data with a cut-off value of 1%; Guibert (2018b) represented the data with a cut-off value of 5%; Guibert (2018c) represented the data with a cut-off value of 10%

As shown in Fig. 4a, four studies involving 209 patients with NSCLC treated with ICI therapy examined the correlation between PD-L1 expression in CTCs and OS. Pooled analysis of these studies indicated no significant association between PD-L1 expression in CTCs and OS (HR = 0.75, 95% CI 0.49–1.13, *P* = 0.170). A moderate level of heterogeneity was observed among studies (*I*^*2*^ = 33.5%, *P* = 0.185). As shown in Fig. 4b, six studies involving 247 patients examined the correlation between PD-L1 expression in CTCs and PFS. The pooled results found that NSCLC patients with PD-L1^+^ CTCs appeared to have a longer PFS than those with negative PD-L1 expression in CTCs, but this difference did not achieve statistical significance (HR = 0.79; 95% CI 0.59–1.06, *P* = 0.12). A low level of heterogeneity was observed between studies (*I*^*2*^ = 20.6%, *P* = 0.266).

**Fig. 5:**
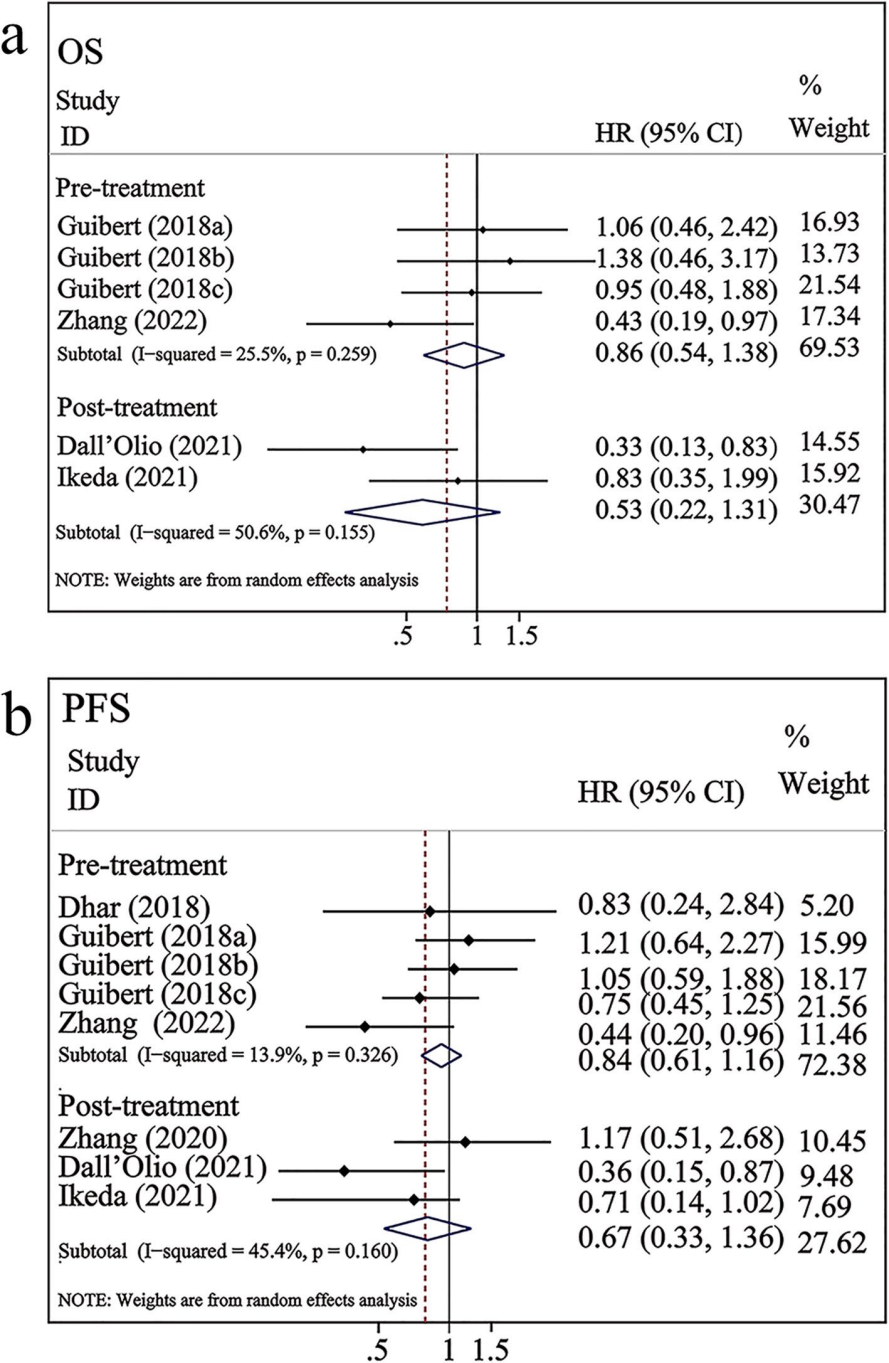
Subgroup analysis of PD-L1 expression in CTCs versus OS/ PFS after different treatment durations

Subgroup analysis of OS and PFS according to time point revealed that PD-L1 expression in CTCs before treatment was linked to a lower HR than PD-L1 expression in CTCs after treatment. Additionally, the pre-treatment or post-treatment PD-L1 expression in CTCs was not significantly correlated with OS (pre-treatment: HR = 0.73, 95% CI 0.44–1.21, *P* > 0.05; post-treatment: HR = 0.83, 95% CI 0.35–1.98, *P* > 0.05; Table 2, Fig. 5a–b) or PFS (pre-treatment: HR = 0.75, 95% CI 0.52–1.08, *P* > 0.05; post-treatment: HR = 0.95, 95% CI 0.50–1.80, *P* > 0.05; Table 2, Fig. 5a–b). Stratified analysis of sample size and region revealed that PD-L1^+^CTCs were associated with superior OS when the sample size was ≤ 30 (HR = 0.43, 95% CI 0.19–0.97, *P* < 0.05; Table 2).

### Correlation between exoPD-L1 and prognosis in patients with NSCLC undergoing ICI therapy

**Fig. 6:**
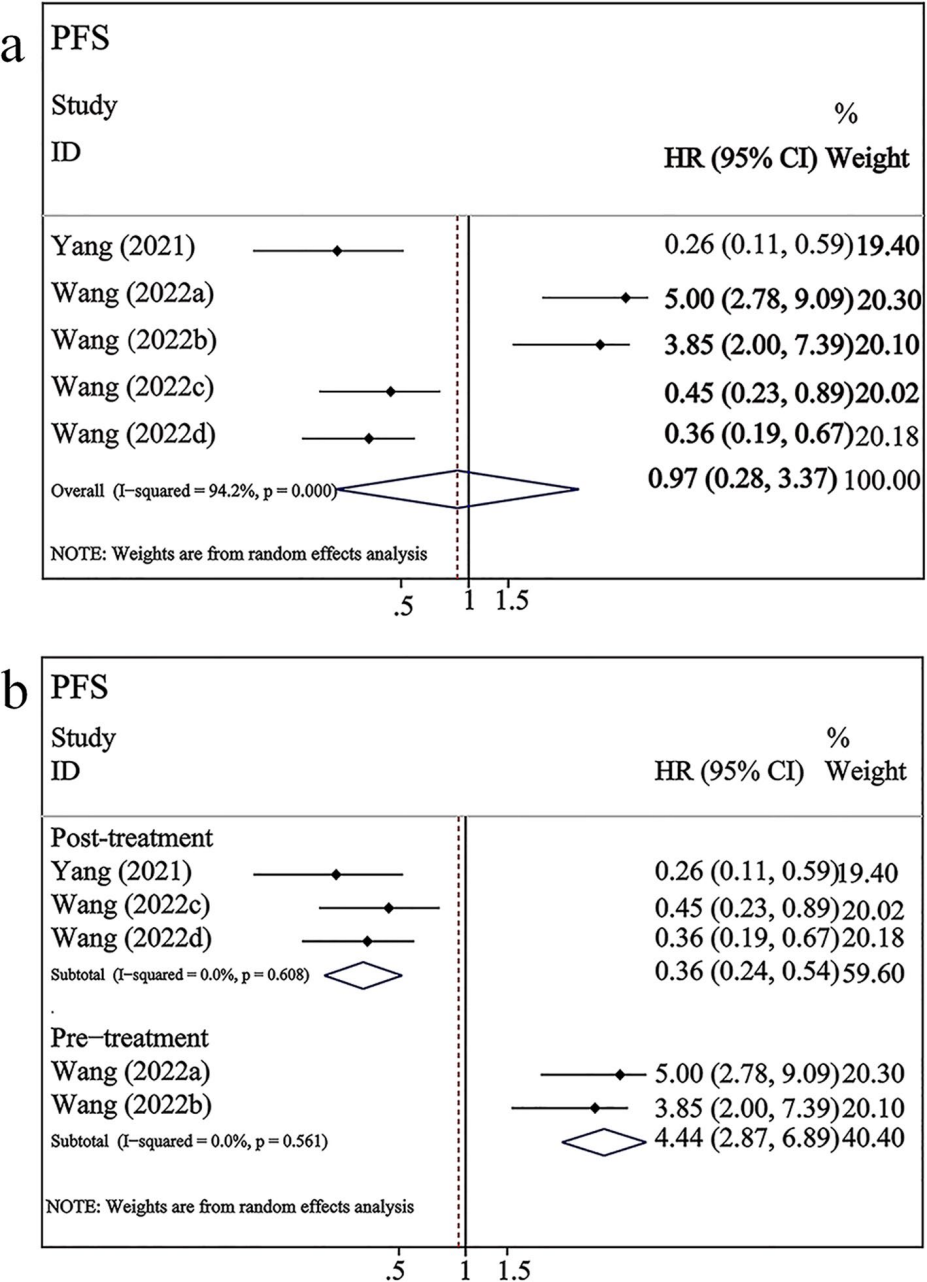
Forest plots of the correlation between exoPD-L1 and PFS in patients undergoing ICI therapy. a: exoPD-L1 expression versus PFS; Wang (2022a) represented the pre-treatment data with mono-immunotherapy; Wang (2022b) represented the pre-treatment data with combination immunotherapy; Wang (2022c) represented the upregulation in the post-treatment data with mono-immunotherapy; Wang (2022d) represented the upregulation in the post-treatment data with combination immunotherapy. b: Subgroup analysis of exoPD-L1 (pre-treatment and post-treatment dynamic changes) and PFS after different treatment durations

Two studies demonstrated the relationship between exoPD-L1 and prognosis in patients with NSCLC undergoing ICI therapy. Of these, one reported the correlation of exoPD-L1 with both OS and PFS, while another involved the correlation with only PFS. As shown in Fig. 6a, two studies involving 170 patients with NSCLC receiving ICI therapy investigated the association between exoPD-L1 and PFS. The overall pooled results showed that exoPD-L1 was not significantly correlated with PFS (HR = 0.97, 95% CI 0.28–3.37, *P* = 0.96), and there was a high level of heterogeneity (*I*^*2*^ = 94.2%, *P* = 0.000). The relationship between the post-treatment dynamic upregulation of exoPD-L1 and OS in patients with NSCLC undergoing ICI therapy was reported only by Yang et al. In particular, the fold-increasing changes of exoPD-L1 were significantly associated with longer OS (HR = 0.20, 95% CI 0.07–0.53, *P* < 0.001).

As studies on the correlation of pre-treatment exoPD-L1 with OS were not retrieved, subgroup analysis was performed with data on exoPD-L1 and PFS after different treatment durations. The results of the subgroup analysis revealed that the difference in treatment duration was the source of heterogeneity. The heterogeneity decreased to 0% in both the pre-treatment and post-treatment subgroups (Fig. 6b). High pre-treatment exoPD-L1 levels were significantly associated with unfavorable PFS (HR = 4.44, 95% CI 2.87–6.89, *P* < 0.001; Fig. 6b). However, post-treatment dynamic exoPD-L1 upregulation was significantly correlated with favorable PFS (HR = 0.36, 95% CI 0.24–0.54, *P* < 0.001; Fig. 6b).

### Correlation between clinicopathological parameters and prognosis in patients with NSCLC undergoing ICI therapy

Five studies [23, 27, 28, 31, 33] involving 511 patients with NSCLC undergoing ICI therapy investigated the association between PFS or OS and clinicopathologic features. In this meta-analysis, six indicators of gender, age, Eastern Cooperative Oncology Group Performance Status (ECOG PS), smoking status, metastatic status, and the expression of tPD-L1 were analyzed in association with PFS or OS. The pooled results indicated that these six indicators do not significantly correlate with PFS and OS in patients with NSCLC (the results are listed in Table S1).

### Publication bias

The Begg's and Egger's tests were performed to examine publication bias. Publication bias was not observed in the three markers (*P* > 0.05) (the *P* values are listed in Table S2). The plots of Begg's and Egger's test results are shown in Fig. S1.

## Discussion

Cancers hide from the immune system through PD-L1 upregulation, which is associated with a poor prognosis [39]. Additionally, the activation of the PD-1/PD-L1 pathway can lead to a series of alterations in the T-cell response, including an enhanced threshold of activated T-cells, reducing their proliferation and promoting their exhaustion and apoptosis [40]. This weakens the anti-cancer immune response, allowing tumors to acquire immune escape capacity. PD-1/PD-L1 pathway inhibitors block the communication between PD-1 and PD-L1, improving the survival of patients with cancer by restoring the immune response against tumor cells [41, 42]. However, the clinical efficacy of ICIs is unsatisfactory, with some patients exhibiting hyper-progression, leading to fatality. Recently, PD-L1 has been widely studied as a promising tumor marker. The tPD-L1 expression level is considered a predictive biomarker for the efficacy of ICIs [43]. However, in patients with advanced NSCLC, performing reproducible detection is challenging as the tumor tissues are difficult to obtain. The emergence of precision medicine has enabled the analysis of molecular biomarkers based on liquid biopsies owing to their portability and reproducibility, which can overcome the spatial and temporal heterogeneity of malignancies. However, the predictive and prognostic values of blood-based PD-L1 indicators in patients with NSCLC undergoing ICI therapy are unclear, warranting a systematic meta-analysis. This is the first systematic and comprehensive analysis of the pre-treatment and post-treatment dynamic changes in the three blood-based PD-L1 biomarkers in patients with NSCLC undergoing ICI therapy. Although some studies have performed meta-analyses of sPD-L1 and PD-L1 in CTCs, they collectively analyzed various tumors [44, 45]. These studies did not individually analyze patients with NSCLC, especially the dynamic changes before and after the application of ICIs. In terms of exoPD-L1, an analogous meta-analysis of exoPD-L1 has not been previously performed. The evaluation of dynamic PD-L1 levels before and after treatment is essential for patients with cancer undergoing ICI therapy. Therefore, it is particularly vital that the levels of blood-based PD-L1 are evaluated at different time points. The strength of this study is that subgroup analyses of the included studies were performed before and after immunotherapy. Meanwhile, the correlation between each marker and prognosis at different treatment times was elucidated.

A review of the literature identified 14 trials with 928 NSCLC cases. In this meta-analysis, seven studies on sPD-L1 involving 554 patients with NSCLC, six studies on PD-L1 in CTCs involving 247 patients with NSCLC, and two studies on exoPD-L1 involving 171 patients with NSCLC were included to investigate the prognostic effect of these blood markers in patients with NSCLC undergoing ICI therapy.

The analysis revealed that high levels of sPD-L1 were significantly correlated with worse OS (HR = 2.11, 95% CI 1.59–2.80, *P* < 0.001) and PFS (HR = 2.14, 95% CI 1.59–2.88, *P* < 0.001). The subgroup analysis results revealed that high pre-treatment levels of sPD-L1 were significantly associated with poorer OS (HR = 2.32, 95% CI 1.68–3.18, *P* < 0.001) and PFS (HR = 2.52, 95% CI 1.72–3.68, *P* < 0.001). However, after immunotherapy, the upregulation of sPD-L1 was not significantly correlated with OS (HR = 1.46, 95% CI = 0.65–3.26, *P* > 0.05) or PFS (HR = 1.62, 95% CI = 0.92–2.86, *P* > 0.05). This result suggests that high pre-treatment levels of sPD-L1 can be considered a biomarker to predict the prognosis of NSCLC patients receiving ICIs. These findings are consistent with those of Han et al., who reported that lower pre-treatment sPD-L1 levels predicted improved PFS and OS [46]. Additionally, another study demonstrated that high baseline levels of sPD-L1 were associated with poor OS in patients who initially received immunotherapy [47]. sPD-L1, a predictor of tumor proliferation, migration, and survival, is reported to be a potential predictive and prognostic biomarker for immunotherapy [48]. However, the dynamic changes in post-treatment sPD-L1 were not associated with prognosis. This meta-analysis included three studies related to post-treatment. Costantini et al. demonstrated that high sPD-L1 levels in NSCLC patients receiving nivolumab were associated with adverse effects and a poorer prognosis [31]. However, Tiako Meyo et al. demonstrated that the upregulated sPD-L1 levels were not significantly associated with PFS and OS in patients treated with nivolumab. Interesting, the authors proposed a composite biomarker, namely the combination of sPD-1 and sPD-L1, to predict the efficacy and prognosis of nivolumab in patients with NSCLC [28]. This provides new insights for future studies. Yang et al. suggested that the dynamic changes of sPD-L1 were not correlated with treatment efficacy and OS but were significantly linked to PD-L1 mRNA and exoPD-L1 levels [26].

Recently, several studies have proposed that high expression of PD-L1 in CTCs is associated with a poorer prognosis in patients with various cancers treated with other therapies, such as chemotherapy or surgery [22, 49, 50]. In contrast, PD-L1 upregulation in CTCs predicts a favorable prognosis in patients with cancer undergoing ICIs [51, 52]. However, the pooled results of this analysis indicated no significant correlation between PD-L1^+^ CTCs and OS (HR = 0.75, 95% CI 0.49–1.13, *P* = 0.170) or PFS (HR = 0.79, 95% CI 0.59–1.06, *P* = 0.12). Furthermore, pre-treatment and post-treatment subgroup analyses did not reveal significant differences. These results suggest that PD-L1^+^ CTCs may not be correlated with the prognosis of patients with NSCLC undergoing ICI therapy. Of the six included studies, three were pre-immunotherapy studies. Guibert et al. demonstrated that the PD-L1^+^ CTCs before immunotherapy were not significantly correlated with prognosis, but this correlation was significant in patients with treatment failure and disease progression [25]. Dhar et al. reported no significant correlation between PD-L1^+^ CTCs before immunotherapy and PFS [35]. This can be attributed to the decreased number of patients included in the study (*n* = 17). However, Zhang et al. demonstrated that pre-treatment PD-L1^+^ CTCs were positively correlated with PFS and OS in patients with NSCLC treated with immunotherapy plus chemotherapy [38]. The results of these two studies were contrasting. We consider that the heterogeneity may be attributable to two factors. First, the treatment protocols were different in the two studies. Guibert et al. utilized nivolumab, whereas Zhang et al. used sintilimab plus docetaxel. Second, the cut-off values of the two studies were markedly different (Table 1). Three studies examined dynamic changes after immunotherapy. Dall'Olio et al. and Ikeda et al. revealed that, compared to patients with negative PD-L1 expression in CTCs, PD-L1^+^ CTCs have significantly longer PFS after immunotherapy [27, 37]. However, Zhang et al. demonstrated that PD-L1 expression in CTCs was not associated with PFS [36].

In this meta-analysis, only two studies related to exoPD-L1 were included. The pooled results showed that elevated pre-treatment exoPD-L1 levels were significantly linked to poorer PFS (HR = 4.44, 95% CI 2.87–6.89, *P* < 0.001; Fig. 4b). However, the dynamic increases in post-treatment exoPD-L1 were associated with superior PFS (HR = 0.36, 95% CI 0.24–0.54, *P* < 0.001) and OS (HR = 0.20, 95% CI 0.07–0.53, *P* < 0.001). In conclusion, the role of exoPD-L1 in determining the prognosis was reversed with the increase in treatment duration. Similarly, one study on solid tumors treated with anti-PD-1 therapy reported that the high pre-treatment exoPD-L1 levels predicted a short PFS [53]. Exosomes belong to one of the subgroups of extracellular vesicles (EVs). EVs, which are lipid-bound vesicles secreted by cells into the extracellular space, comprise the following three primary subtypes: microvesicles (MVs), exosomes, and apoptotic bodies. These subtypes can be differentiated based on their release pathways, dimensions, contents, and functions [54–56]. Tumor cells can utilize EVs to evade immune responses and promote cancer progression and metastasis [57]. Additionally, tumor cells can secrete a large amount of PD-L1 through EVs. ExoPD-L1 binds to PD-1 through its extracellular domain, and the removal of exoPD-L1 can inhibit tumor growth [58]. Chen et al. found that exoPD-L1 can interact with activated T-cells [59]. Circulating exoPD-L1 was closely associated with cancer progression and immune suppression in patients with cancer. This is consistent with the findings of some reviews [60]. However, elevated exoPD-L1 levels after several treatment cycles predicted a good prognosis in patients with NSCLC. Similarly, some studies found that the higher the level of exoPD-L1 increased after applying PD-1 antibodies, the longer the prognosis of cancer patients [24, 26, 59]. Several studies have also confirmed an increase in tPD-L1 expression in the early stages of treatment in patients who respond to ICIs [61]. During the early stages of immunotherapy, PD-L1 expression in tissues and exoPD-L1 in circulation may be upregulated. This is because immunotherapy blocks the interaction between PD-1 and PD-L1, leading to the release of most PD-L1 through exosomes. Therefore, increased PD-L1 expression after immunotherapy may exert a positive immune regulatory effect. However, de Miguel-Perez et al. found that the dynamic increase of EV PD-L1 in the blood of patients with NSCLC was strongly associated with treatment failure and decreased survival [62]. This finding is in contrast to that of this study. The reason for the heterogeneity can be attributed to two factors. First, Yang et al. and Wang et al. detected exoPD-L1 using the Simoa platform and enzyme-linked immunosorbent assay, respectively. However, de Miguel-Perez et al. used the immunoblotting method for the detection of EV PD-L1. Second, two studies included in this meta-analysis analyzed PD-L1 in the exosomes, whereas de Miguel-Perez et al. analyzed PD-L1 in EV. Recent studies have confirmed that PD-L1 can be detected in both exosomes and MVs [59, 63]. Exosomes are only one subtype of EVs, and the effect of MVs on the prognosis has not been previously reported. In addition, Del Re et al. evaluated the relationship between PD-L1 mRNA in plasma-derived exosomes and the efficacy of ICIs in patients with NSCLC. The results showed that the upregulation of exosomal PD-L1 mRNA was significantly and negatively associated with treatment efficacy [63]. Notably, recent studies have suggested that the PD-L1 mRNA level is distinct from the protein level, owing to post-translational regulatory mechanisms [58, 64].

### Limitations

This meta-analysis has several limitations. First, the number of included studies and the sample size were small. Small-sample studies may overestimate the results of efficacy and prognostic evaluations. Second, the pooled results revealed that post-treatment sPD-L1 levels and PD-L1 expression in CTCs were not correlated with the prognosis of patients with NSCLC undergoing ICI therapy. This can be attributed to the high heterogeneity among the studies. Most studies have no definite consensus on the cut-off values of biomarkers and utilize various assay platforms, which may have contributed to the high heterogeneity of these studies and biased outcomes. Therefore, a criterion threshold must be established through a uniform and standardized protocol. In the future, the correlation of PD-L1 expression in CTCs and post-treatment sPD-L1 levels with the prognosis of NSCLC patients receiving ICI therapy needs to be validated using large-sample and multi-center clinical trials. Third, only three indicators were included in the analysis. EVs and some immune cells in the blood also contain PD-L1, such as dendritic cells and myeloid-derived suppressor cells [65], which were not included in this meta-analysis. In the following work, we plan to systematically analyze all blood-based PD-L1 markers and apply more analytical tools such as bioinformatic analysis and Mendelian randomized trials to screen valuable signature genes. Fourth, most of the included studies analyzed only individual markers but not multi-target or multi-gene combinations. Furthermore, these three markers were not validated in clinical trials. In the future, clinical studies combining multiple markers will be performed to validate the characteristic markers.

## Conclusions

The comprehensive meta-analysis of three blood-based PD-L1 indicators suggested that pre-treatment sPD-L1 and exoPD-L1 serve as unfavorable prognostic factors in patients with NSCLC undergoing ICI therapy. In contrast, the dynamic upregulation of post-treatment exoPD-L1 levels indicates a favorable prognosis. However, post-treatment sPD-L1 levels and PD-L1 expression in CTCs were not correlated with the prognosis of patients with NSCLC undergoing ICI therapy. The blood-based PD-L1 analysis can provide clinicians with a convenient biomarker and complement tPD-L1 analysis to improve the clinical application of ICIs. It is also a potential strategy for predicting treatment efficacy and prognosis in patients with cancer.

### Supplementary Information

**Additional file 1.** PRISMA Checklist.

**Additional file 2.** Full search strategies.

**Additional file 3:** **Table S1.** Correlation of OS/PFS and clinicopathological characteristic of NSCLC patients. **Table S2.** Results of Begg's and Egger's tests of 3 indicators related to PFS/OS. **Fig.S1.** Results of Begg's and Egger's tests of 3 indicators related to PFS/OS a, Beeg’s and Egger’s between sPD-L1 and OS; b, Beeg’s and Egger’s between sPD-L1 and PFS; c, Beeg’s and Egger’s between PD-L1 in CTCs and OS; d, Beeg’s and Egger’s between PD-L1 in CTCs and PFS; e, Beeg’s and Egger’s between exoPD-L1 score and PFS.

## Data Availability

The data analyzed during the current study are available from the corresponding author.

## Abbreviations

NSCLC: Non-small cell lung cancer
SCLC: Small cell lung cancer
sPD-L1: Soluble PD-L1
exoPD-L1: Exosomal PD-L1
HR: Hazard ratio
CI: Confidence interval
OS: Overall survival
PFS: Progression-free survival
ICIs: Immune checkpoint inhibitors
PD-1: Programmed cell death protein 1
PD-L1: Programmed cell death ligand 1
CTLA-4: Cytotoxic T lymphocyte antigen 4
NOS: Newcastle-Ottawa Scale
ECOG PS: Eastern Cooperative Oncology Group Performance Status
EVs: Extracellular vesicles
MVs: Microvesicles
