# Supplementary material for: Prognostic significance of blood-based PD-L1 analysis in patients with non-small cell lung cancer undergoing immune checkpoint inhibitor therapy: a systematic review and meta-analysis
Source: World J Surg Oncol. 2023 Oct 11;21:318. doi: 10.1186/s12957-023-03215-2 (PMC10566159; doi:10.1186/s12957-023-03215-2)
Supplement: Supplementary file 2 — Additional file 2. Full search strategies. [file 12957_2023_3215_MOESM2_ESM.doc]

1. **Pubmed database search strategy**

**Search conditions: All fieles /Title/Abstract/Mesh**

(“Lung cancer” OR “Lung tumor” OR “lung neoplasms” OR “non-small cell lung cancer” OR “non-small cell lung carcinoma” ) AND (“ Immune checkpoint inhibitor” OR “PD-1 inhibitor” OR “PD-L1 inhibitor” OR “antiPD1” OR “antiPD-L1” OR “programmed death‐1 receptor” OR “ programmed death ligand‐1” OR “ cytotoxic T lymphocyte antigen‐4” OR “CTLA-4 inhibitor”) AND (“Peripheral blood” OR “serum” OR “biomaker” OR “soluble PD-L1” OR “sPD-L1” OR “exosomal PD-L1”OR “circulating tumor cells” OR “exoPD-L1” OR “CTCs” OR “PD-L1 in CTCs” OR “blood PD-L1” OR “Circulating PD-L1” OR “soluble programmed death ligand-1” OR “exosomal programmed death ligand-1”)

1. **Embase database search strategy**

**Search conditions: Title /Abstract**

'lung cancer':ab,ti OR 'lung tumor':ab,ti OR 'lung neoplasms':ab,ti OR “non-small cell lung cancer” :ab,ti OR “non-small cell lung carcinoma”) AND 'immune checkpoint inhibitor':ab,ti OR 'pd-1 inhibitor':ab,ti OR 'pd-l1 inhibitor':ab,ti OR ' programmed death‐1 receptor ':ab,ti OR ' programmed death ligand‐1':ab,ti OR 'cytotoxic t lymphocyte antigen 4 inhibitor':ab,ti OR 'ctla 4':ab,ti) AND ' Peripheral blood ':ab,ti OR ' biomaker ':ab,ti OR' soluble PD-L1':ab,ti OR' sPD-L1':ab,ti OR ' exosomal PD-L1 ':ab,ti OR ' exoPD-L1 ':ab,ti OR' circulating tumor cells ':ab,ti OR' CTCs ':ab,ti OR' PD-L1 in CTCs ':ab,ti OR' blood PD-L1 ':ab,ti OR' Circulating PD-L1':ab,ti ' soluble programmed death ligand-1':ab,ti OR' exosomal programmed death ligand-1 ':ab,ti

1. **Cochrane database search strategy**

**Search conditions: Title /Abstract/ Keyword**

(“Lung cancer” OR “Lung tumor” OR “lung neoplasms” “non-small cell lung cancer” OR “non-small cell lung carcinoma” ) AND (“ Immune checkpoint inhibitor” OR “PD-1 inhibitor” OR “PD-L1 inhibitor” OR “antiPD1” OR “antiPD-L1” OR “programmed death‐1 receptor” OR “ programmed death ligand‐1” OR “ cytotoxic T lymphocyte antigen‐4” OR “CTLA-4 inhibitor”) AND (“Peripheral blood” OR “serum” OR “biomaker” OR “soluble PD-L1” OR “sPD-L1” OR “exosomal PD-L1”OR “circulating tumor cells” OR “exoPD-L1” OR “CTCs” OR “PD-L1 in CTCs” OR “blood PD-L1” OR “Circulating PD-L1” OR “soluble programmed death ligand-1” OR “exosomal programmed death ligand-1”)
