# Supplementary material for: Prognostic significance of blood-based PD-L1 analysis in patients with non-small cell lung cancer undergoing immune checkpoint inhibitor therapy: a systematic review and meta-analysis
Source: World J Surg Oncol. 2023 Oct 11;21:318. doi: 10.1186/s12957-023-03215-2 (PMC10566159; doi:10.1186/s12957-023-03215-2)
Supplement: Supplementary file 3 — Additional file 3: Table S1. Correlation of OS/PFS and clinicopathological characteristic of NSCLC patients. Table S2. Results of Begg's and Egger's tests of 3 indicators related to PFS/OS. Fig.S1. Results of Begg's and Egger's tests of 3 indicators related to PFS/OS a, Beeg’s and Egger’s between sPD-L1 and OS; b, Beeg’s and Egger’s between sPD-L1 and PFS; c, Beeg’s and Egger’s between PD-L1 in CTCs and OS; d, Beeg’s and Egger’s between PD-L1 in CTCs and PFS; e, Beeg’s and Egger’s between exoPD-L1 score and PFS. [file 12957_2023_3215_MOESM3_ESM.doc]

Table S1. Correlation of OS/PFS and clinicopathological characteristic of NSCLC patients

| **Features** | **N** | **HR (95% CI)** | **Interaction (*P*)** | **Heterogeneity** |
| --- | --- | --- | --- | --- |
| **OS** |  |  |  | **I^2^%, *P*** |
| Sex (female vs male) | 2 | 0.98 (0.71–1.36) | 0.992 | 0%, 0.737 |
| Age (<median vs ≥median) | 3 | 0.99 (0.94–1.03) | 0.591 | 0%, 0.492 |
| ECOG PS (≥ 2 vs 0–1) | 3 | 1.71 (0.85–3.44) | 0.136 | 41.5%, 0.181 |
| Smoking status (current or former/never) | 3 | 1.04 (0.67–1.62) | 0.862 | 0%, 0.730 |
| Liver\bone\brain metastasis (presence vs. absence) | 4 | 0.96 (0.48–1.93) | 0.918 | 55.7%, 0.081 |
| tPD-L1 (≥ 1% vs <1%) | 5 | 0.99 (0.96–1.03) | 0.619 | 61.0%, 0.036 |
| **PFS** |  |  |  |  |
| Sex (female vs male) | 2 | 0.69 (0.48–1.01) | 0.051 | 0%, 0.572 |
| Age (<median vs ≥median) | 3 | 0.99 (0.96–1.02) | 0.570 | 0%, 0.492 |
| ECOG PS (≥ 2 vs 0–1) | 3 | 1.20 (0.74–1.94) | 0.454 | 0%, 0.659 |
| Smoking status (current or former/never) | 4 | 1.12 (0.86–1.45) | 0.412 | 0%, 0.715 |
| Liver\bone\brain metastasis (presence vs. absence) | 4 | 1.29 (0.83–2.02) | 0.258 | 11.3%, 0.336 |
| tPD-L1 (≥ 1% vs <1%) | 6 | 0.99 (0.95–1.04) | 0.723 | 78.1%, 0.000 |

Aberrations: ECOG, Eastern Cooperative Oncology Group Performance Status; tPD-L1, tissue programmed cell death ligand 1; OS, overall survival; PFS, progression-free survival.

Table S2. Results of Begg's and Egger's tests of 3 indicators related to PFS/OS.

| **Begg’s** | | **Egger’s** |
| --- | --- | --- |
| **P values** | | **P values** |
| **sPD-L1** |  |  |
| OS | 0.602 | 0.341 |
| PFS | 0.548 | 0.414 |
| **PD-L1 in CTCs** |  |  |
| OS | 1 | 0.769 |
| PFS | 0.771 | 0.467 |
| **exoPD-L1** |  |  |
| PFS | 0.462 | 0.291 |

Aberrations: OS, overall survival; PFS, progression-free survival; sPD-L1, soluble PD-L1; exoPD-L1, exosomal PD-L1; PD-L1 in CTCs, programmed cell death ligand 1 in circulating tumor cells.


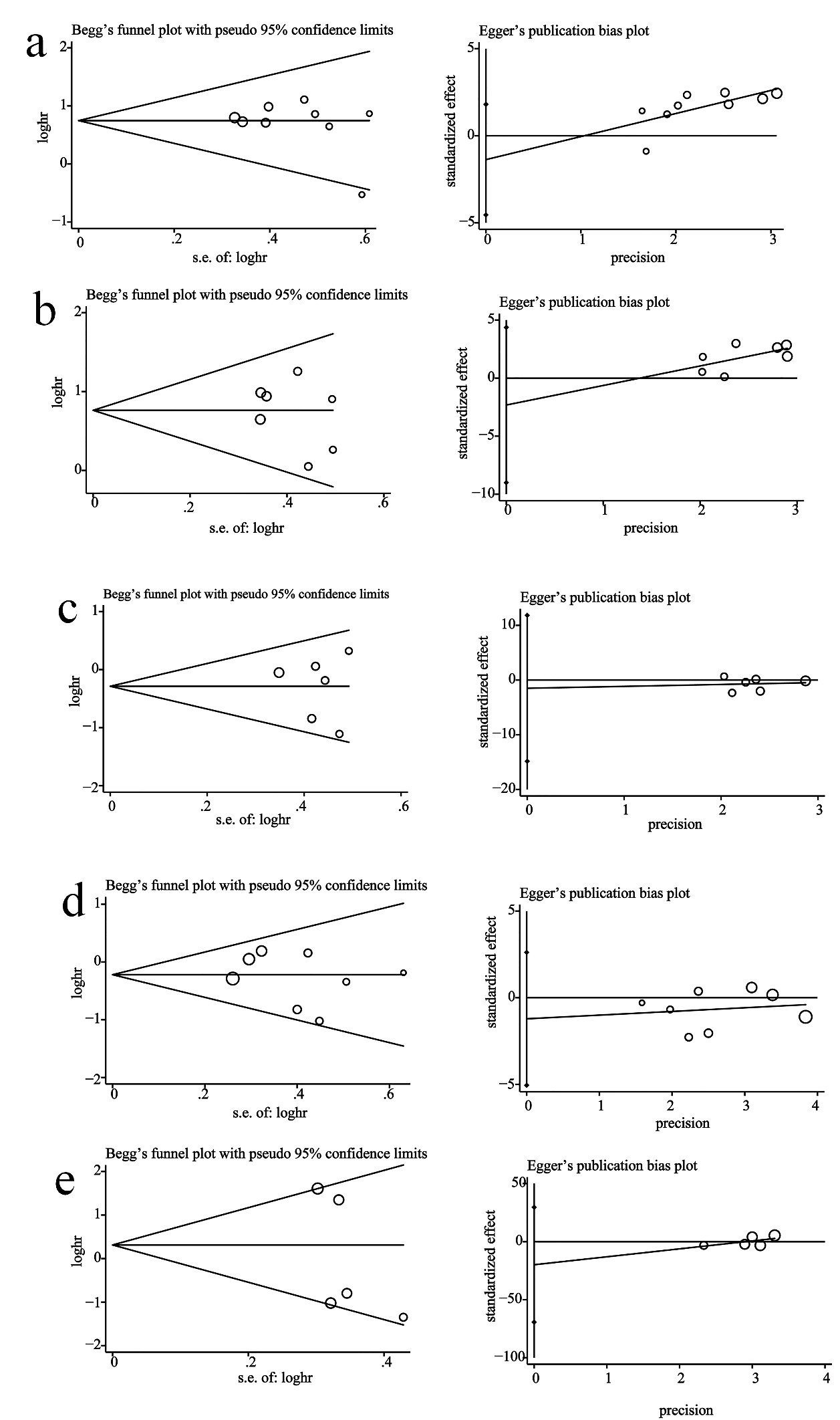


Fig.S1. Results of Begg's and Egger's tests of 3 indicators related to PFS/OS a, Beeg’s and Egger’s between sPD-L1 and OS; b, Beeg’s and Egger’s between sPD-L1 and PFS; c, Beeg’s and Egger’s between PD-L1 in CTCs and OS; d, Beeg’s and Egger’s between PD-L1 in CTCs and PFS; e, Beeg’s and Egger’s between exoPD-L1 score and PFS.
